# Close to recommended caloric and protein intake by enteral nutrition is associated with better clinical outcome of critically ill septic patients: secondary analysis of a large international nutrition database

**DOI:** 10.1186/cc13720

**Published:** 2014-02-10

**Authors:** Gunnar Elke, Miao Wang, Norbert Weiler, Andrew G Day, Daren K Heyland

**Affiliations:** 1 Department of Anaesthesiology and Intensive Care Medicine, University Medical Centre Schleswig-Holstein, Campus Kiel, Arnold-Heller-Strasse 3 Haus 12, 24105 Kiel, Germany; 2 Clinical Evaluation Research Unit, Kingston General Hospital, Angada 4, K7L 2V7, Kingston, ON, Canada

## Abstract

**Introduction:**

Current international sepsis guidelines recommend low-dose enteral nutrition (EN) for the first week. This contradicts other nutrition guidelines for heterogenous groups of ICU patients. Data on the optimal dose of EN in septic patients are lacking. Our aim was to evaluate the effect of energy and protein amount given by EN on clinical outcomes in a large cohort of critically ill septic patients.

**Methods:**

We conducted a secondary analysis of pooled data collected prospectively from international nutrition studies. Eligible patients had a diagnosis of sepsis and/or pneumonia and were admitted to the ICU for ≥3 days, mechanically ventilated within 48 hours of ICU admission and only receiving EN. Patients receiving parenteral nutrition were excluded. Data were collected from ICU admission up to a maximum of 12 days. Regression models were used to examine the impact of calorie and protein intake on 60-day mortality and ventilator-free days.

**Results:**

Of the 13,630 patients included in the dataset, 2,270 met the study inclusion criteria. Patients received a mean amount of 1,057 kcal/d (14.5 kcal/kg/day) and 49 g protein/day (0.7 g/kg/d) by EN alone. Patients were mechanically ventilated for a median of 8.4 days and 60-day mortality was 30.5%. An increase of 1,000 kcal was associated with reduced 60-day mortality (odds ratio (OR) 0.61; 95% confidence interval (CI) 0.48 to 0.77, *P* <0.001) and more ventilator-free days (2.81 days, 95% CI 0.53 to 5.08, *P* = 0.02) as was an increase of 30 g protein per day (OR 0.76; 95% CI 0.65 to 0.87, *P* <0.001 and 1.92 days, 95% CI 0.58 to 3.27, *P* = 0.005, respectively).

**Conclusions:**

In critically ill septic patients, a calorie and protein delivery closer to recommended amounts by EN in the early phase of ICU stay was associated with a more favorable outcome.

## Introduction

Critically ill patients are characterized by marked variations in energy requirements [1,2] and net protein catabolism due to disproportional cytokine and stress hormone release [3-5]. These patients are prone to develop energy and protein deficits over their time of ICU stay, as they are unable to resume oral feeds. To prevent adverse outcomes related to nutritional deficits such as increased infectious complications, nutritional support is warranted [6-8]. Current guidelines uniformly recommend enteral nutrition (EN) as first-line therapy, starting early within 24 to 48 hours after ICU admission [9-11]. Early EN has thereby several non-nutritional benefits such as supporting the immune and metabolic responses as well as preserving gut integrity [12]. However, it is still less clear what the optimal dose of EN should be, particularly during the first week of illness, and how specific subgroups of critically ill patients respond to the amount of energy and protein delivered by EN.

Three recent large studies [13-15] have compared full enteral feeding to intentional underfeeding or trophic nutrition, that is provision of small volume EN aiming to produce positive local effects on gastrointestinal mucosa and beneficial systemic effects [16]. None of these studies showed an effect on mortality [13-15]. Based on these studies, the updated Surviving Sepsis Campaign guidelines [17] suggest to avoid mandatory full caloric feeding but use low-dose enteral feeding in the first week of ICU stay (evidence grade 2B). This contradicts the 2013 Canadian critical care nutrition guidelines that recommend optimizing the dose of EN and not using an initial strategy of trophic feeds for five days [18] based on randomized but also large-scale observational studies in heterogeneous critically ill patients [19-22].

Given the paucity of prospective randomized clinical trials in septic patients and in view of the contradictory recommendations, we conducted a secondary analysis of a large international nutrition database. The aim was to evaluate the effect of energy and protein intake given by EN on clinical outcomes in a large cohort of critically ill septic patients receiving only EN. We hypothesized that EN delivery of calories and protein closer to recommended amounts in the early phase of critical illness would be associated with increased survival and ventilator-free days.

## Material and methods

### Study design and patients

This retrospective study was a secondary analysis of pooled data from the International Nutrition Survey (INS) and baseline data from the Enhanced Protein-Energy Provision via the Enteral Route in Critically Ill Patients (PEP uP) study [23,24]. Data were prospectively collected from 737 ICUs in 33 countries annually during five study periods between 2007 and 2011. Institutional ethics approval was obtained from the Health Sciences Research Ethics Board at Queen’s University, Kingston, ON as the responsible ethics committee and all other participating sites were advised and agreed to contact their local institutional ethics boards regarding the necessity of ethics approval for study participation. The need for informed patient consent was waived given the nature of the studies (for PEP uP: a system-level quality improvement study and INS: an observational survey).

Details of the study design, data collection and management were described previously [23,24]. In these studies, critically ill adult patients who were mechanically ventilated within the first 48 hours of ICU admission and with an ICU stay more than three days were enrolled. This *a priori* removed potentially confounding patients that have short stays in ICU (less than three days), receive little EN, and universally have a good outcome [25]. The current analysis was restricted to those critically ill adult patients with an ICU admission diagnosis of sepsis and pneumonia, given that mechanically ventilated patients admitted with pneumonia are most often septic [26,27]. The Acute Physiology and Chronic Health Evaluation II (APACHE II) admission diagnosis taxonomy was used to codify the admission diagnosis. This case determination was not adjudicated but abstracted from a chart by the health-care professional responsible for collecting the data of the concerned nutrition study. In addition, only those patients who were receiving exclusively EN were included. We thereby intended to preclude possible confounding effects of parenteral nutrition (PN) on the amount of nutrition as PN has a treatment effect different and distinct from EN [28-30]. Site characteristics, patients’ baseline demographic and physiologic data, and severity of illness were collected at the time of study enrollment. Daily information on nutrition therapy including the amount of nutrition received by EN (calories and protein), morning serum glucose levels, and use of promotility drugs were collected daily for a maximum of 12 days or until death or ICU discharge. Nutrition therapy was left at the discretion of each site investigator as no study-specific standardized nutrition protocol was followed.

ICU and hospital outcomes, including ventilation status, length of ICU and hospital stays, and mortality, were determined from hospital records at 60 days after ICU admission.

### Statistical analysis

Site characteristics are presented as means and ranges or counts and percentages. Patient characteristics, nutritional variables and clinical outcomes are presented as counts and percentages for categorical variables, medians and quartiles for ventilator-free days and length of stay variables, and means and standard deviations for other continuous variables.

Nutrition adequacy was defined as the total amount of energy or protein received from EN over the first 12 ICU days divided by the amount prescribed at baseline and expressed as a percentage. Days after permanent progression to exclusive oral feeding were excluded, but days without EN prior to exclusive oral feeding were counted as 0.

Logistic and linear regression with random intercepts to account for within ICU dependence were used to associate mortality and ventilator-free days respectively to energy and protein (in separate models) received during the first 12 ICU days prior to death or permanent switch to exclusive oral feeding. Energy and protein were modeled separately due to their high co-linearity. The models were run without adjustment and adjusting for days with nutrition, body mass index (BMI), age and APACHE score. In accordance with a previous publication [23], we report the odds of 60-day mortality and the expected mean change in ventilator-free days separately per increase of 1,000 kcal and 30 g of protein, respectively. In order to aid in the interpretability of these models, we report the relationship between nutritional intake and 60-day mortality by dividing patients into groups of tertiles based on the amounts of energy and protein received and using regression techniques to describe subsequent effect on outcome. This tertile grouping (based on distribution of intake) is consistent with what previous investigators have published in the literature [25,31]. In addition, we performed a sensitivity analysis to examine the association between EN intake during only the first seven days of ICU stay and subsequent outcome in a subset of patients. To be eligible for this analysis, patients had to receive at least seven days of EN and be alive for subsequent assessment of mortality. Patients who died before day seven or who were transferred out of ICU or transition to oral feeds prior to day seven were excluded from this sensitivity analysis. This selection strategy better separating exposure (nutrition intake in the first seven days) from outcome assessment (subsequent mortality) is consistent with the time period (first seven days) the Surviving Sepsis campaign guidelines [17] refer to.

Statistical analyses were performed using SAS software, version 9.2 (SAS Institute, Cary, NC, USA).

## Results

### Site and patient characteristics

**Figure 1:**
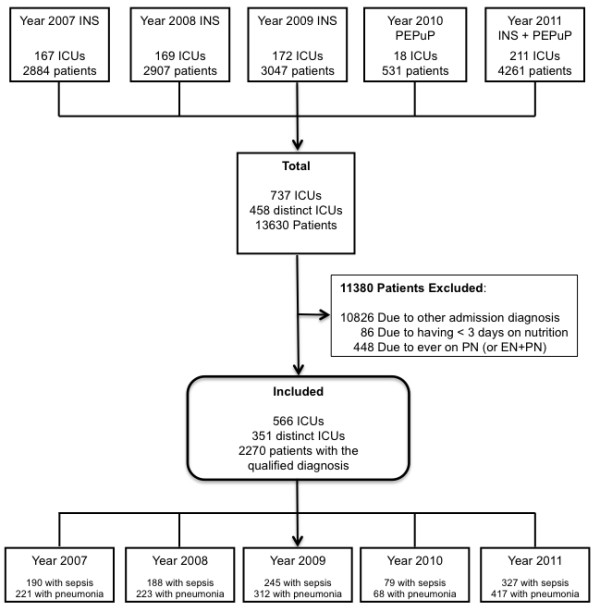
**Flow chart of study population.** Of 13,630 patients enrolled in the nutrition surveys and PEP uP trial between 2007 and 2011, 2,270 patients with pneumonia and sepsis from 351 ICUs were included in the final analysis. The 411 patients from the 2007 database were already included in a previous study [23]. EN, enteral nutrition; ICU, intensive care unit; INS, International Nutrition Survey; PN, parenteral nutrition.

A detailed flowchart showing the flow of patients in this analysis is given in Figure 1. Of 13,630 patients enrolled in the surveys, 2,270 patients from 351 ICUs were included in the current analysis. Forty-five percent of these patients had sepsis and 55% had pneumonia. The majority of ICUs (77.8%) had an implemented feeding protocol including a protocol for glycemic control as well as dietitian(s) present (84.1%) (Table A1 in Additional file 1). The mean age of the patients was 61.7 years, 56.3% were male and mean BMI was 27.6. Nearly half of the patients (45.9%) had a BMI below 25 kg/m^2^. Mean APACHE II score was 23.9 (Table 1).

**Table 1:** Characteristics and clinical outcomes of study patients

**Number of patients**	**Total**
	**n = 2,270**
Age, years (SD)	61.7 (17.0)
Sex, male (%)	1,277 (56.3)
Height, metres (SD)	1.68 (0.11)
Weight, kg (SD)	78.1 (26.0)
BMI, mean (SD)	27.6 (8.3)
BMI, n (%)	
<24	1,039 (45.9)
25-29	594 (26.2)
30-40	463 (20.4)
>40	169 (7.5)
Admission type	
Medical, n (%)	2,270 (100)
Surgical	0
ICU admission diagnosis, n (%)	
Aspiration pneumonia	330 (14.5)
Bacterial/viral pneumonia	911 (40.1)
Sepsis (other than urinary tract)	859 (37.8)
Sepsis of urinary tract origin	170 (7.5)
APACHE II score, mean (SD)	23.9 (7.9)
Days from hospital admission to ICU admission (hours), median [IQR]	6.55 [0.08, 65.08]
Days from hospital admission to ICU admission, n (%)	
[0–1] day	1,437 (63.4)
[1-3] days	303 (13.4)
>3 days	527 (23.3)
Length of ICU stay (days), median [IQR]	11.5 [6.9-21.4]
Length of hospital stay (days), median [IQR]	23.8 [13.8-48.5]
Length of mechanical ventilation (days), median [IQR]	8.4 [4.6-19.2]
Ventilator-free days, median [IQR]	46.4 [4.0-55.0]
Mortality (60 day),%	30.5

Length of ICU stay, hospital stay and mechanical ventilation were based on 60-day survivors only. SD, standard deviation; BMI, body mass index; ICU, intensive care unit; APACHE II, Acute Physiology and Chronic Health Evaluation II; IQR, interquartile range.

### Nutrition therapy

Comprehensive information on nutrition therapy is described in Table 2. On average, EN was started within 48 hours (mean ± standard deviation (SD) 26.6 ± 26.4 hours from admission to ICU). Overall, study patients received a mean energy of 1,057 ± 480 kcal/day (14.5 ± 7.2 kcal/kg/day) resulting in 61% adequacy of calories from prescribed EN. Mean protein intake was 49 ± 24 g/day (0.7 ± 0.3 g/kg/day), representing 57% adequacy of protein prescription. The progression of calories and protein by EN over the first 12 ICU days is shown in Figure A1 in Additional file 2. The weight-based formula was found to be the most frequent method to calculate energy requirements while indirect calorimetry was only rarely used. The actual body weight was predominantly used for calculation of nutrition prescription (50.4% of patients). Mean morning serum glucose level was 7.9 mmol/l. In 451 patients who ever had EN interrupted due to high gastric residual volumes, motility agents were used in 68.1% of these patients while only 12.6% received small bowel feeding. Arginine-supplemented and fish oil-supplemented formulas were used in only 3.5% and 6.3% of study patients, respectively (Table 2).

**Table 2:** Nutrition data of study patients

**Number of patients**	** Total**
	**n = 2,270**
Nutritional prescription	
Mean energy, kcal/day (SD)	1,757.7 (352.1)
Mean energy, kcal/kg/day (SD)	23.9 (5.7)
Mean protein, gram/day (SD)	88.2 (24.7)
Mean protein, gram/kg/day (SD)	1.2 (0.3)
Nutrition received	
Mean energy, kcal/day (SD)	1,056.9 (480.5)
Mean energy, kcal/kg/day (SD)	14.5 (7.2)
Adequacy of calories from nutrition therapy, % (SD)	60.8 (26.3)
Mean protein, gram/day (SD)	48.9 (24.3)
Mean protein, gram/kg/day (SD)	0.7 (0.3)
Adequacy of protein from nutrition therapy, % (SD)	57.0 (26.6)
Number of patients on nutrition therapy, n (%)	
EN only	2,204 (97.1)
None	66 (2.9)
Hours to initiation of EN mean (SD)	26.6 (26.4)
Mean morning blood glucose (mmol/l) (SD)	7.9 (1.9)
Head of elevation	32.7 (9.9)
Motility agent use in patients with EN intolerance*, n (%)	307 (68.1)
Small bowel feeding in patients with EN intolerance*, n (%)	57 (12.6)
EN formula, n (%)	
Arginine-enriched formula	79 (3.5)
Fish oil-enriched formula	143 (6.3)
Glutamine-enriched formula	5 (0.2)
Polymeric formula	1,976 (87.1)
Body weight used in calculation of nutrition prescription, n (%)	
Actual body weight	1,144 (50.4)
IBW based on Hamwi formula	117 (5.2)
IBW based on BMI 20–25 kg/m^2^	254 (11.2)
Other	691 (30.4)
No weight used in calculation	18 (0.8)
Missing	46 (2.0)
Method used to calculate energy requirements^#^, n (%)	
Indirect calorimetry	13 (0.6)
Harris-Benedict equation	259 (11.4)
Weight-based formula	1,220 (53.7)
Other^§^	854 (37.6)
None	8 (0.4)

*Among 451 patients who were ever on EN and ever had feeds interrupted due to high gastric residual volumes; ^#^sum of patient numbers within this category may be higher than total column patient number since one patient could contribute to multiple methods in the PEPuP and INS 2011 studies; ^§^other includes Schofield with/without adjustment for stress and activity, Ireton-Jones, Mifflin-St. Jeor equation and fixed calorie target. kcal, kilocalories; SD, standard deviation; EN, enteral nutrition; IBW, ideal body weight; BMI, body mass index.

### Outcomes

Clinical outcomes of the whole study patient population are shown in Table 1 and the outcomes of the patients included in the sensitivity analysis are shown in Table A2 in Additional file 1. In the whole population, overall mortality was 30.5% at 60 days. Median length of mechanical ventilation was 8.4 days with a length of stay in the ICU of 11.5 days. Table 3 and Table A3 in Additional file 1 present the relationship between increased nutrition and mortality or ventilator-free days, respectively, for both the entire study population and the sensitivity analysis. Both adjusted and unadjusted analyses revealed that the provision of each additional 1,000 kcal per day was associated with significant reduction in 60-day mortality (adjusted odds ratio (OR) 0.61, 95% confidence interval (CI) 0.48 to 0.77) and an increase in ventilator-free days (adjusted 2.81 days, 95% CI 0.53 to 5.08) as was the provision of additional 30 g protein per day (adjusted OR 0.76, 95% CI 0.65 to 0.87 and 1.92 days, 95% CI 0.58 to 3.27, respectively). The 60-day mortality results remained virtually unchanged when we estimated the effect of only the first seven days of EN on subsequent mortality among patients who were alive and evaluable for nutritional support for at least seven days. However, the positive effect on ventilator-free days only remained statistically significant with an increased protein but not caloric intake (see Table A3 in Additional file 1). In addition, Table A4 in Additional file 1 shows that the lowest tertile of energy and protein intake received per day (patients receiving ≤865 kcal/d and ≤39.5 g/d, respectively) was associated with increased 60-day mortality as compared to the highest tertile (patients receiving ≥1,294 kcal/d and ≥58.9 g/d, respectively).

**Table 3:** Relationship between enteral nutrition and 60-day mortality

	**Unadjusted**			**Adjusted**		
	**Odds ratio**	**95% CI**	***P *** **value**	**Odds ratio**	**95% CI**	***P *** **value**
**A: Total study population (n = 2,270)**						
**Energy intake**						
Per 1,000 kcal	0.51	(0.41-0.64)	<0.001	0.61	(0.48-0.77)	<0.001
**Protein intake**						
Per 30 gram	0.70	(0.61-0.80)	<0.001	0.76	(0.65-0.87)	<0.001
**B: Sensitivity analysis (n = 1,560)**						
**Energy intake**						
Per 1,000 kcal	0.56	(0.44-0.71)	<0.001	0.61	(0.48-0.79)	<0.001
**Protein intake**						
Per 30 gram	0.72	(0.62-0.83)	<0.001	0.75	(0.64-0.87)	<0.001

Odds of 60-day mortality per increase of 1,000 kilocalories (top) and 30 gram of protein (bottom) received per day both unadjusted and adjusting for nutrition days, BMI, age, and APACHE II score. Panel A shows data in the total study population and Panel B the data for the patients included in the sensitivity analysis who received enteral nutrition at least 7 days in the ICU and who were alive and evaluable for subsequent outcome. CI, confidence interval; kcal, kilocalories.

## Discussion

This secondary analysis of a large nutrition database included 2,270 patients with sepsis and pneumonia with an ICU length of stay ≥3 days. The analysis was deliberately restricted to patients receiving EN alone to avoid the possible confounding effect of PN and specifically address the relationship between the amount of EN and outcome. The main finding was that an increased amount of calories and protein per day during the early phase of ICU stay was associated with lower 60-day mortality and an increase in ventilator-free days. Our findings should not be interpreted that we recommend overfeeding but rather, that the amount of calories and protein better approximates that which was prescribed.

Our results are in contrast to the findings from three recent prospective randomized studies that compared full enteral feeding to intentional underfeeding or trophic nutrition and did not show a significant mortality difference [13-15]. Two most recently published follow-up analyses of the largest, the so-called EDEN trial revealed no differences in long-term physical or cognitive outcomes six and twelve months after initial trophic or full enteral feeding [32,33]. Based on these studies, the updated Surviving Sepsis Campaign guidelines suggest to avoid full caloric feeding and use low-dose enteral feeding in the first week of ICU stay (evidence grade 2B) [17]. Perhaps the differences between these studies and our analysis could be explained by the differences in patient populations studied. The study by Arabi *et al*. [13] only included a limited number of septic patients (72 of a total of 240 patients). The EDEN trial [15] included a selected sample of patients with acute lung injury that were relatively young (mean age 52 years), well-nourished and fairly obese (average BMI of around 30) whereas our population was older (average age 62 years) and almost half of the patients had a low to normal BMI. In a prior analysis, Alberda and colleagues [23] demonstrated that patients with a BMI from 25 to 35 may not be sensitive to differing amounts of EN, at least with respect to mortality, whereas a mortality reduction was associated with receiving more EN in patients with BMI <25 and >35. A further point is that the average duration of ICU stay in the EDEN study was five days compared to eleven days in our study. To the extent that early targeted EN is particularly relevant in patients with longer length of stay [34,35], this may further explain the discordant results. All of these observations and explanations lead us to question the generalizability of the EDEN study [15] to practice, which is best represented by our study population reflecting a more heterogenous septic population representative of real-life settings.

Our findings are consistent with the Canadian clinical practices guidelines that do not recommend trophic feeds or intentional undernourishment [18], which are based on prospective randomized but also large observational trials in other, non-septic patients. Two of these were randomized controlled studies showing that patients with enhanced enteral feeding had subsequent decreased infection, hospital length of stay, and a trend toward reduced mortality, respectively, compared to patients receiving standard nutrition with lower amounts of nutrition [21,22]. Two observational studies, one in 207 medical-surgical ICU patients [36] and the other in 103 critically ill patients with burns [37] consistently showed that those who enterally received greater amounts of energy and protein had significantly lower infectious morbidity or mortality, respectively. Randomized controlled trials are considered to represent most valid evidence to inform clinical guidelines. However, for many reasons, as outlined by Vincent [38] they cannot persistently provide conclusive answers. Evidence from well-conducted cohort studies may provide additional relevant and valid information [39,40] and should be taken into account for clinical guideline implementation, as they are closer to the real daily nutritional practice in the ICU setting [41].

In this respect, the major strength of our study is the large number of septic patients enrolled from multiple, international ICUs, which is to our knowledge the largest report on nutrition therapy in this critically ill patient population. Observing the same signals as Alberda *et al*. [23], who included all patients admitted to the ICU not just septic patients, and given the large and heterogenous group of critically ill septic patients from many sites. enhances the likelihood that our data are valid. The statistical analysis used was robust by adjusting for timing, length of nutrition and ICU stay as well as severity of illness. We thereby avoided a potential bias of incorrect adjustment for patients with short ICU stays that receive very little nutrition and typically have a very good outcome. The inability or inadequate nature of adjusting for these low-risk patients is largely the reason why some studies show that better fed patients have worse clinical outcomes [25]. We also used a sensitivity analysis to examine the association between nutritional intake during the first seven ICU days and outcome.

Admittedly, our results should still be interpreted with caution and viewed as hypothesis generating given the pooled observational nature of the study. Thus, the link between better delivery of calories and protein (providing closer to recommended amounts) and better outcomes may not be a causal relationship. It could be that less sick patients tolerate their nutrition better and independently have a better clinical outcome. Furthermore, we are unable to categorize our patients with respect to the level of sepsis (severe sepsis or septic shock) because these data to grade severity of illness were not collected in our dataset. A further limitation is that our results are not generalizable to surgical critically ill patients with sepsis as only medical patients were represented in our study.

## Conclusions

In conclusion, our study showed that a closer to recommended EN intake (that is calories and protein) given during the early phase of ICU stay was associated with a more favorable outcome in critically ill septic patients. These findings disagree with three recent prospective randomized trials on which the current Surviving Sepsis Campaign guidelines base their recommendation to use low-dose rather than full enteral feeding in septic patients. Further large trials are warranted based on our hypothesis-generating results that septic patients at high nutrition risk benefit from early increased EN.

## Key messages

• A daily calorie and protein delivery closer to recommended amounts by EN within the early phase of ICU stay was associated with shorter duration of mechanical ventilation and lower mortality in septic patients.

• These findings challenge the current Surviving Sepsis Campaign guidelines that recommend the use of low-dose rather than full enteral feeding in septic patients.

• Further large trials are warranted based on these hypothesis-generating results that septic patients at high nutrition risk benefit from early increased EN.

## Abbreviations

APACHE II: Acute Physiology and Chronic Health Evaluation II; BMI: body mass index; CI: confidence interval; EN: enteral nutrition; IBW: ideal body weight; ICU: intensive care unit; INS: International Nutrition Survey; kcal: kilocalories; OR: odds ratio; PN: parenteral nutrition; SD: standard deviation.

## Competing interests

GE has received speaker’s honoraria from Abbott, B Braun, and Fresenius Kabi. DKH has received research grants and speaker honorarium from Fresenius Kabi, Baxter, and Biosyn. All other authors declare that they have no competing interests.

## Authors’ contributions

GE and DKH conceived the study and drafted the manuscript. DKH, MW and AGD performed the statistical analysis. GE, MW, NW, AGD and DKH participated in the analysis and interpretation of data and revision of the manuscript. All authors read and approved the final manuscript.

## Supplementary Material

Additional file 1: Table A1 Characteristics of participating ICUs. **Table A2.** Clinical outcomes of patients included in the sensitivity analysis. **Table A3.** Relationship between enteral nutrition and ventilator-free days. **Table A4.** Relationship between enteral nutrition (tertile groups) and 60-day mortality). Click here for file

Additional file 2 Progression of calories and protein by enteral nutrition. Click here for file
