# Supplementary material for: Close to recommended caloric and protein intake by enteral nutrition is associated with better clinical outcome of critically ill septic patients: secondary analysis of a large international nutrition database
Source: Crit Care. 2014 Feb 10;18(1):R29. doi: 10.1186/cc13720 (PMC4056527; doi:10.1186/cc13720)
Supplement: Additional file 1: Table A1 — Characteristics of participating ICUs. Table A2. Clinical outcomes of patients included in the sensitivity analysis. Table A3. Relationship between enteral nutrition and ventilator-free days. Table A4. Relationship between enteral nutrition (tertile groups) and 60-day mortality). [file cc13720-S1.doc]

**Close to recommended caloric and protein intake by enteral nutrition is associated with better clinical outcome of critically ill septic patients: Secondary analysis of a large international nutrition database**

Gunnar Elke, Miao Wang, Norbert Weiler, Andrew G. Day, Daren K. Heyland

**Additional file 1**

**Additional Tables**

**Table A1. Characteristics of participating ICUs**

|  |  | **ICUs included in analysis** |
| --- | --- | --- |
|  | Number of distinct ICUs | N=351 |
| **Hospital Type** | |  |
|  | Teaching | 260 (74.1%) |
|  | Non-teaching | 91 (25.9%) |
|  |  |  |
| **Size of Hospital (beds)** | |  |
|  | mean (range) | 591 (99-3000) |
| **Multiple ICUs in hospital** | |  |
|  | Yes | 205 (58.4%) |
|  | No | 146 (41.6%) |
| **ICU Structure** | |  |
|  | Open | 102 (29.1%) |
|  | Closed | 243 (69.2%) |
|  | Other | 6 (1.7%) |
| **Presence of Medical Director** | |  |
|  | Yes | 332 (94.6%) |
|  | No | 19 (5.4%) |
| **Size of ICU (beds)** | |  |
|  | mean (range) | 17.6 (4-75) |
| **ICU has Feeding Protocol** | |  |
|  | Yes | 273 (77.8%) |
|  | No | 78 (22.2%) |
| **Presence of Dietitian(s)** | |  |
|  | Yes | 295 (84.1%) |
|  | No | 56 (15.9%) |
| **Full Time Equivalent Dietitian (per 10 beds)** | | |
|  | mean (range) | 0.6 (0-6.9) |
| **Glycemic Control Protocol*** | |  |
|  | Yes | 306 (87.2%) |
|  | No | 45 (12.8%) |

ICU, intensive care unit

218 ICUs participated one year only, 154 ICUs participated two years, 99 ICUs participated three years, 80 ICUs participated four years, and 15 ICUs participated 5 years. ICUs that participated in more than one year are reported using the most recent responses.

*As this was an observational study, local clinical practice was not influenced but simply recorded whether or not a glycemic control protocol was being used at each site. Thus, there were likely differences in target ranges of glucose control across the sites.

Table A2. Clinical outcomes of patients included in the sensitivity analysis

| Number of Patients | N=1673 |
| --- | --- |
| Length of ICU stay (days)  Median [IQR] | 15.7 [10.2-27.5] |
| Length of hospital stay (days)  Median [IQR] | 25.6 [15.3-47.3] |
| Length of mechanical ventilation (days)  Median [IQR] | 12.0 [7.8-22.7] |
| Mortality % (60 day) | 31.6 |
| Ventilator-free days  Median [IQR] | 40.3 [3.3-52.1] |

ICU, intensive care unit; IQR, interquartile range; Und, undefined

Clinical outcomes of patients receiving enteral nutrition at least 7 days in the ICU and who were alive and evaluable for subsequent mortality.

Length of ICU stay, hospital stay and mechanical ventilation were based on 60-day survivors only.

**Table A3. Relationship between enteral nutrition and ventilator-free days**

**A: Total study population (n=2270)**

**Energy Intake**

|  | Unadjusted | | | Adjusted | | |
| --- | --- | --- | --- | --- | --- | --- |
|  | Beta | 95% CI | *P* Value | Beta | 95% CI | *P* Value |
| Per 1000 kcal | 4.81 | (2.54-7.08) | <0.001 | 2.81 | (0.53-5.08) | 0.02 |

**Protein Intake**

|  | Unadjusted | | | Adjusted | | |
| --- | --- | --- | --- | --- | --- | --- |
|  | Beta | 95% CI | *P* Value | Beta | 95% CI | *P* Value |
| Per 30 gram | 2.91 | (1.57-4.26) | <0.001 | 1.92 | (0.58-3.27) | 0.005 |

**B: Sensitivity analysis (n=1560)**

**Energy Intake**

|  | Unadjusted | | | Adjusted | | |
| --- | --- | --- | --- | --- | --- | --- |
|  | Beta | 95% CI | *P* Value | Beta | 95% CI | *P* Value |
| Per 1000 kcal | 3.71 | (1.23-6.18) | 0.003 | 2.28 | (-0.20-4.77) | 0.07 |

**Protein Intake**

|  | Unadjusted | | | Adjusted | | |
| --- | --- | --- | --- | --- | --- | --- |
|  | Beta | 95% CI | *P* Value | Beta | 95% CI | *P* Value |
| Per 30 gram | 2.56 | (1.08-4.05) | <0.001 | 1.85 | (0.36-3.34) | 0.02 |

CI, confidence interval

Beta estimates expected increase in ventilator-free days per 1000 kcalories (top) and 30 gram of protein (bottom) per day both unadjusted and adjusting for nutrition days, BMI, age, and APACHE II score. For Beta point estimate expected increase in ventilator-free days from linear regression 0 is the null - and thus confidence intervals crossing 0 are not significant.

Panel A shows data in the total study population and Panel B the data for the patients included in the sensitivity analysis who received enteral nutrition at least 7 days in the ICU and who were alive and evaluable for subsequent outcome.

# Table A4. Relationship between enteral nutrition (tertile groups) and 60-day mortality

Energy Intake

|  | n | Unadjusted | | | Adjusted | | |
| --- | --- | --- | --- | --- | --- | --- | --- |
|  |  | Odds Ratio | 95% CI | *P* Value | Odds Ratio | 95% CI | *P* Value |
| **Energy Intake** |  |  |  | <0.001 |  |  | <0.001 |
| ≤865 kcal/d | 756 | 1.88 | (1.46-2.41) |  | 1.59 | (1.22-2.07) |  |
| (865, 1294) kcal/d | 757 | 1.13 | (0.90-1.43) |  | 1.00 | (0.79-1.28) |  |
| ≥1294 kcal/d | 756 | ref |  |  | ref |  |  |

Protein Intake

|  | n | Unadjusted | | | Adjusted | | |
| --- | --- | --- | --- | --- | --- | --- | --- |
|  |  | Odds Ratio | 95% CI | *P* Value | Odds Ratio | 95% CI | *P* Value |
| **Protein Intake** |  |  |  | <0.001 |  |  | <0.001 |
| ≤39.5 g/d | 757 | 1.83 | (1.42-2.35) |  | 1.64 | (1.26-2.13) |  |
| (39.5, 58.9) g/d | 757 | 1.25 | (0.99-1.58) |  | 1.16 | (0.91-1.48) |  |
| ≥58.9 g/d | 755 | ref |  |  | ref |  |  |

CI, confidence interval; n, number of patients; g/d, gram per day; kcal/d, kcalories per day; ref, reference

Odds of 60-day mortality per tertile increase of energy intake (upper diagram) and protein (lower diagram) received per day both unadjusted and adjusting for nutrition days, BMI, age, and APACHE II score.
