# Supplementary material for: Close to recommended caloric and protein intake by enteral nutrition is associated with better clinical outcome of critically ill septic patients: secondary analysis of a large international nutrition database
Source: Crit Care. 2014 Feb 10;18(1):R29. doi: 10.1186/cc13720 (PMC4056527; doi:10.1186/cc13720)
Supplement: Additional file 2 — Progression of calories and protein by enteral nutrition. [file cc13720-S2.doc]

**Close to recommended caloric and protein intake by enteral nutrition is associated with better clinical outcome of critically ill septic patients: Secondary analysis of a large international nutrition database**

Gunnar Elke, Miao Wang, Norbert Weiler, Andrew G. Day, Daren K. Heyland

**Additional file 2**

**Figure A1. Progression of calories and protein by enteral nutrition**

**A**

**B**

ICU, intensive care unit; N, number of patients

Panel A shows the progression of calories and Panel B the progression of protein received by enteral nutrition as a percentage of the maximum calories/protein prescribed over the first 12 ICU days. The number of patients included per day is reported below each diagram.
